# Editorial: Understanding the role of new media in psychiatry

**DOI:** 10.3389/fpsyt.2025.1553205

**Published:** 2025-01-17

**Authors:** Lucie Kalisova, Jozef Buday

**Affiliations:** ^1^ Department of Psychiatry, First Faculty of Medicine, Charles University, Prague, Czechia; ^2^ Department of Psychiatry, Military Hospital in Prague, Prague, Czechia

**Keywords:** digital media, mental health, sexual victimization, stigma, electroconvulsive therapy, virtual reality, Youtube

New media mediate interpersonal contact via digital technologies. Users access all content through computers, smartphones, and tablets. The rise of new media has increased communication between people all over the world. Since the Covid pandemic, the role of new media strengthened. New media undoubtedly brings many advantages to psychiatry, such as internet-based psychotherapeutic programs, virtual reality training, and many others, and allows the availability of mental health specialists via online communication. New media, on the other hand, can influence users’ mental health negatively. Incorporation of digital technologies into the lives of users can increase addictive behavior and cause social isolation. Social media content can be harmful, and virtual relationships substitute real ones. This Research Topic highlights various aspects of the role of new media in psychiatry.

The article by Orsolini et al. focused on sexual violence facilitated by digital technologies. Social interactions are no longer only in person but increasingly occurring on virtual platforms (social media, internet). People can be exposed to virtually mediated interpersonal violence, which can cause trauma, resulting in a decrease in well-being and even triggering mental health problems (anxiety, depression, suicidality, and others). The prevalence of technologically facilitated sexual violence (for example sextortion, cyberbullying, sexual harassment, cyberstalking) is significant (1). The only assessment tool for an adult population available is Technology Facilitated Sexual Violence – Victimization Scale (TFSV-VS) (2). The study of Orsolini et al. presents the development and validation of an Italian version of TFSV-VS. In the second part of their study, they used this questionnaire to evaluate technology-facilitated sexual violence in a sample of 223 Italian youths, and the results revealed a high proportion of the sample experiencing digital sexual and gender/sexuality harassment. In the previous context, it is clear that sexual victimization via digital technologies has to be taken into account more seriously.

The Review of Buday et al. provides an overview of depiction of electroconvulsive therapy (ECT) in video games. The negative stigma connected to electroconvulsive therapy has been kept in the public mind until today in spite of the modernization of this treatment method, its superior effectivity, and the minimalization of side effects. Historical sources of the stigma included different media – mainly film and press. According to a review by Sienaert, movies, TV programs, and sitcoms (1948-2016) described a negative image of ECT as an obsolete, damaging treatment method torturing humans with (and also without) mental illness, and the image has not improved over time (3). There are over 3 billion gamers worldwide, so the description of ECT in this media type is definitely influencing public image (4). Even though not many games reflect ECT, unfortunately, the portrait of ECT in video games confirms a negative image of ECT.

Virtual reality has become a tool for the assessment and treatment of various mental illnesses in the last decades. VR is effectively used in the treatment of anxiety disorders and PTSD and also as a training tool for preserving cognitive abilities in dementia or improving social skills in autism spectrum disorders (5). The technical quality of VR is increasing every year, allowing accurate immersion and, thus, a subjective feeling of presence in a virtual situation. A systematic review of Antos et al. focuses on proper alignment between virtual and real hands. They included articles assessing healthy subjects’ factors that can influence the discrepancy between the virtual and actual limb position (positional offset), which can have an impact on immersion and the presence of users. Identifying influencing factors is important to ensure therapeutic approaches have the highest possible user presence.

The article of Choi et al. from South Korea tried to explore whether there is a relation between YouTube video characteristics and viewer’s mental health traits. Many teenagers and young adults spend a lot of time on this video sharing platform, and the addictive potential of such digital platforms has been repeatedly proven (6). The authors developed algorithms which enable to assess violence, color saturation and brightness in YouTube videos. These characteristics were related to self- assessment of level of stress, depression and anxiety in YouTube users. This study is more like a pilot study, the sample was small, but it was the first study focused on the influence of YouTube content on mental health characteristics.

This Research Topic concerns a significant and recent problem. As the extent of new media increases in society, research interest in this topic will rise quickly.
